# What Makes Paediatric Patients So Much at Risk of Medical Adhesive‐Related Skin Injury in Intensive Care Unit?

**DOI:** 10.1111/nicc.70082

**Published:** 2025-06-03

**Authors:** Özlem Şensoy, Seda Çağlar, Emine Aybı, Seher Erdoğan

**Affiliations:** ^1^ Department of Pediatric Nursing, School of Nursing Istanbul University Istanbul Turkey; ^2^ Department of Pediatric Nursing, Florence Nightingale Faculty of Nursing Istanbul University‐Cerrahpaşa Istanbul Turkey; ^3^ Department of Pediatric Intensive Care Umraniye Training and Research Hospital İstanbul Turkey

**Keywords:** adhesive, medical adhesive‐related skin injuries, nursing, paediatric, paediatric intensive care

## Abstract

**Background:**

Medical adhesives are frequently used during hospitalization to secure medical devices to the skin. Compared to adults, children are at a higher risk of developing medical adhesive‐related skin injuries (MARSI).

**Aim:**

The aim of this study is to determine the incidence, characteristics and associated risk factors of MARSI in a Paediatric Intensive Care Unit (PICU).

**Study Design:**

This prospective cohort study included 72 children who were followed from November 10, 2023, to February 10, 2024, from admission until the onset of MARSI or discharge. The risk of skin injury was assessed using the Glamorgan Scale, and patients' sociodemographic and clinical data were obtained from medical records.

**Results:**

The incidence of MARSI was 61.1%, with 44 out of 72 patients developing MARSI, resulting in a total of 140 cases. The majority of cases were skin stripping (72.1%). The median time to MARSI occurrence was 2.78 days. Being 12 months old or younger (OR: 5.3, 95% CI: 1.6–17.6, *p* = 0.006), an increasing number of medical devices (OR: 2.4, 95% CI: 1.1–5.2, *p* = 0.026) and sedation use for more than 24 h (OR: 5.4, 95% CI: 1.7–16.8; *p* = 0.003) were identified as independent risk factors.

**Conclusions:**

This study revealed a high incidence of MARSI in the PICU. MARSI develops rapidly in the early days of hospitalization. Critically ill patients aged 12 months or younger, with a greater number of medical devices and prolonged sedation, are at a higher risk of developing MARSI.

**Relevance to Clinical Practice:**

The high incidence of MARSI underscores the need for proactive prevention strategies. Skin assessment for MARSI should be conducted before, during and at each change of medical adhesive applications. Patients aged 12 months or younger, patients with an increased number of medical devices, and patients undergoing prolonged sedation require greater attention to MARSI prevention.

**Summary**

- What is known about the topic
  ○ Medical adhesives are commonly used in health care settings to secure devices to the skin.
  ○ Children are at a higher risk than adults for developing medical adhesive‐related skin injuries (MARSI) due to differences in skin structure and sensitivity.
  ○ MARSI is a preventable complication that can occur during hospitalization, particularly in intensive care settings.
- What this paper adds
  ○ The incidence of MARSI in a Paediatric Intensive Care Unit is as high as 61.1%.
  ○ The most common type of medical adhesive‐related skin injuries is skin stripping, and it occurs within the first few days after the initial medical adhesive application.
  ○ Being 12 months old or younger, having an increasing number of medical devices and prolonged sedation increase the risk of developing MARSI.

## 1 Introduction and Background

Medical adhesives are widely used to secure medical devices to the skin, especially in intensive care units [1]. MARSI, characterized by skin damage following the removal of medical adhesives (MAs), can cause discomfort, pain, delayed healing and infection, particularly in children with fragile skin [1, 2, 3, 4, 5].

Children treated in the intensive care unit are particularly vulnerable to skin injuries in the presence of underlying health conditions such as impaired tissue perfusion, neurological changes, oedema and immobility [1, 3, 4, 6]. In a study conducted in a paediatric intensive care unit (PICU) in South Korea, the prevalence of MARSI was reported to be 58.3%, and it was found that the most common type of injury was skin stripping and the risk of MARSI increased especially in cases requiring prolonged use of adhesives such as central venous catheter dressings or ETT fixation [7]. In a PICU in China, the incidence of MARSI was found to range from 25.53% to 54.17%, and the majority of these injuries were identified as skin stripping and skin tears. Additionally, being younger than 2 years, the presence of oedema, prolonged hospital stays and infections were reported as risk factors for the development of MARSI [4]. Another study conducted in a paediatric cardiac intensive care unit in Brazil reported an incidence of MARSI of 60.3% in children, with younger age, the number of medical devices used, duration of mechanical ventilation and length of ICU stay identified as risk factors for MARSI development [8]. Although MARSI is a common problem in the paediatric population, it is still under‐recognized.

The literature emphasises that reducing the incidence of MARSI can prevent patient pain and discomfort, shorten hospital stays and reduce health care costs [1, 2, 5, 9]. The fact that MARSI is preventable and has a direct impact on the quality of care makes its management critical [1, 2, 10, 11, 12].

Paediatric nurses are responsible for monitoring and caring for patients, choosing appropriate fixation materials and preventing and reducing complications. Therefore, understanding the incidence, characteristics and risk factors of MARSI is important to prevent these injuries in paediatric patients. This study aims to determine the incidence, characteristics (classification, location and type of MA used) and risk factors associated with MARSI in the PICU.

### 1.1 Aim and Research Questions

The study aimed to determine the incidence and associated risk factors of MA‐related skin injury (MARSI) in a PICU.

The research questions were as follows:

- What are the incidence rates and associated risk factors of MARSI in a PICU?
- What are characteristics of MARSI in a PICU?

## 2 Design and Methods

This research was planned as a prospective cohort study.

### 2.1 Setting and Sample

This study was conducted in the PICU of a training and research hospital in Turkey between November 10, 2023 and February 10, 2024. The patients aged 18 years and younger admitted to the PICU are consecutively enrolled in the study. The exclusion criteria were as follows: (1) existing MARSI before admission to the PICU (2) death before the follow‐ups were completed, (3) discharge and readmission during follow‐up time and (4) skin disease (e.g., epidermolysis bullosa and Steven‐Johnson syndrome).

### 2.2 Data Collection Tools

#### 2.2.1 The Descriptive Information Form

This form was developed by the researchers based on the current literature [1, 4, 7, 8] and consisted of 17 questions including sociodemographic (age, sex, body mass index), mobilization status, Glamorgan score, number of medical devices and clinical characteristics (duration of hospitalization, diagnosis, comorbidities, respiratory support, nutritional status, use of sedation, use of vasopressor medications, use of corticoids, mobilization status, laboratory values). These data were retrieved from the electronic medical record by two investigators (ŞÖ, EA).

#### 2.2.2 MARSI Monitoring Form

The researchers were developed this form to collect data on MARSI during each dressing chancing or removal. The form includes the date of first the first application of the dressing, the date of dressing change, the MAs used the fix any medical devices, the development of MARSI, characteristics (classification, location and type of medical adhesive used) of the MARSI (if a MARSI developed). McNichol et al.'s (2013) classification of MARSI (skin stripping, skin tears, dermatitis and others) was used for classification [11].

#### 2.2.3 Glamorgan Paediatric Pressure Ulcer Risk Assessment Scale

This scale was developed by Willock, Baharestani and Anthony (2007) for the diagnosis of paediatric patients aged 0–18 years. The Turkish validity and reliability study was conducted by Saçar et al. in 2013 [13, 14]. The scale is composed of nine items: (1) mobility, (2) equipment/devices/hard surface pressing or rubbing the skin, (3) significant anaemia, (4) persistent fever, (5) impaired peripheral perfusion, (6) inadequate nutrition, (7) low serum albumin (< 35 g/L), (8) weight below the 10th percentile and (9) incontinence. Each item is scored based on the present impairment, and these nine scores are added to obtain a total score ranging from 0 to 42 points. The higher the score, the greater the impairment and, consequently, the higher the risk of developing PI. According to the scale criteria, there are three risk stratifications for the total score: at risk (10–14), high risk (15–19) and very high risk (20+).

### 2.3 Data Collection

Data were collected by two principal investigators (ŞÖ, EA), who are an academic nurse and the head nurse of the PICU. The principal investigator (ŞÖ) who is experienced in skin assessment, identification of MARSIs and all study procedures trained the other investigator (AE) to improve the types of familiarity with MARSIs and to help better identify this classification of skin injury when one occurs. The training, consisting of two 30‐min slide presentations with MARSI photos, focuses on MARSI risk factors and MARSI assessment. Subsequently, each PI assessed patients (they assessed the same patients) hospitalized in the PICU for 1 week as a pilot study to determine the consistency of the analysis. In the clinic where the study was conducted, care requiring adhesive change was performed as a daily routine at a certain time interval. In case of adhesive change outside this routine, bedside nurses informed the investigators and the skin was evaluated by the investigators (ŞÖ, AE). The MARSI Monitoring Form was used during the observations. PIs also photographed the skin and any MARSIs they detected (if occurred) at each adhesive change. In case of difficulty in classifying the injury, an external wound care nurse identified and classified the injury for consistency. The follow‐up sessions were terminated when a MARSI was detected. During the follow‐up period of the patients, previous MARSI that occurred at the same site and was used for the same purpose were excluded from the total number of MARSIs. However, a MARSI occurring at a new site during the evaluation process was recorded as a new case. Patients' descriptive information and Glamorgan paediatric pressure ulcer risk scores were obtained during each follow‐up session. Pilot study data were not included in the analysis.

### 2.4 Outcomes

The primary aim of the study was to determine the incidence of MARSI in the PICU. Secondary outcomes were to identify characteristics of MARSI, including its classification, location, the type of medical adhesive used and associated risk factors.

### 2.5 Data Analysis

The data were analysed using the SPSS software, version 26.0 (Statistical Package for Social Sciences, IBM Corp., Armonk, NY, USA). The Kolmogorov Smirnov test was used to test the normality. Categorical variables were summarized as frequencies and percentages (*n*, %), whereas continuous variables were expressed as mean and standard deviation (mean ± SD). Comparisons of continuous variables between two independent groups (participants who developed MARSI vs. those who did not) were performed using the Mann–Whitney *U* test due to the non‐normal distribution of the data. Chi‐square tests (Pearson chi‐square and Fisher's exact test) were used to compare categorical data. The logistic regression analysis was used to identify the risk factors of MARSI. The ROC (receiver operating characteristic) curve analysis was used for the optimal number of medical devices to predict the occurrence of MARSI. The Youden index was used to determine the optimal cut‐off value of the medical device. In the study, three different incidence rates were calculated (1) per 100 patients, (2) per 100 medical adhesive (MA) and (3) per 1000 days of MA use: The formula used to calculate each incidence rate was (1) incidence rate per 100 patients = number of MARSIs/number of patients × 100, (2) incidence rate per 100 MAs = number of MARSIs/number of MAs × 100 and (3) incidence rate per 1000 days of MA use = number of MARSIs/days of MA usage × 1000. The relationship between the variables was considered statistically significant when *p* < 0.05 (two sided). The STROBE checklist was used for reporting the study [15].

### 2.6 Ethical and Research Approvals

The study received ethical approval from the Ümraniye Training and Research Hospital Ethics Committee on 2/11/2023 (Reference No: B.10.1.TKH.4.34.H.GP.0.01/435). Additionally, written institutional permission was obtained from the institution where the research was conducted. Parents or guardians of children included in the study were informed about the research, voluntary informed consent forms were signed and consent was obtained from applicable children. The research was conducted in accordance with the principles of the Helsinki Declaration.

## 3 Results

### 3.1 Descriptive Information of Children

**FIGURE 1:**
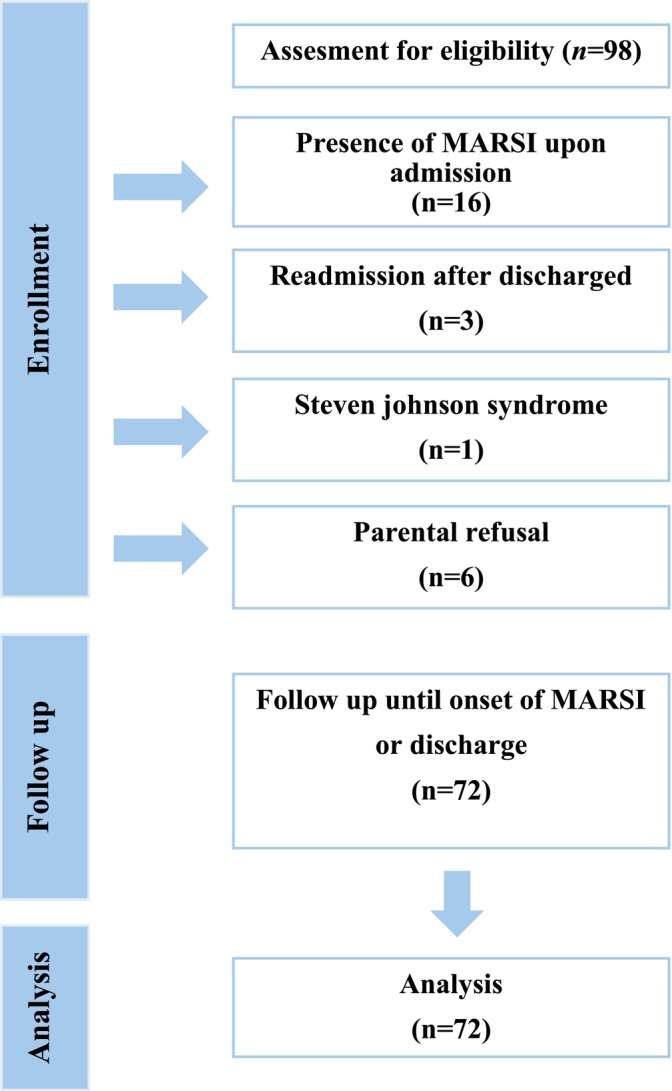
Study flow chart.

Total of 98 patients were screened for eligibility, but 16 were excluded due to the presence of MARSI upon admission, 3 were readmissions after discharge, 1 had Steven‐Johnson Syndrome and 6 were not enrolled due to parental refusal for study participation. The data were analysed based on 72 patients (Figure 1). The descriptive characteristics of participants are shown in Table 1. Forty (56%) of the patients were boys and 32 (44%) were under 1 year of age. A considerable proportion (49%) of the patients were hospitalized due to respiratory system diseases. The mean duration of hospitalization was 8.04 (SD = 5.18) days.

**TABLE 1:** Descriptive information of patients (*N* = 72).

Parameters	Category	Total
Age, *n* (%)	< 1 year	32 (44.4)
	≥ 1 year	40 (55.6)
Sex, *n* (%)	Male	40 (55.6)
	Female	32 (44.4)
BMI, mean (SD)	All	15.6 (4.3)
Hospital stay (day), mean (SD)	All	8.04 (5.18)
Diagnosis, *n* (%)	RD	35 (48.6)
	ND	11 (15.3)
	GID	15 (20.8)
	CD	5 (6.9)
	CA^5^	6 (8.3)
Mobilization status, *n* (%)	Immobilized	46 (63.9)
	Mobile/Semi‐Restricted	26 (36.1)
Glamorgan score, mean (SD)	All	21.7 (6.9)
Number of medical devices, mean (SD)	All	4.2 (1.3)

*Note:* The values are the means ± SDs or *n* (%).

Abbreviations: BMI, body mass index; CA, cancer; CD, cardiovascular diseases; GID, gastrointestinal diseases; ND, neurological diseases; RD, respiratory disease.

### 3.2 Incidence Rate of MARSI

The incidence rate of MARSI was calculated as 61.1. Totally 140 cases of MARSI occurred in 44 patients using a total of 302 MAs over 1869 days. The incidence of MARSI was calculated as 61.1 cases per 100 patients, 14.6 cases per 100 MAs and 23.5 cases per 1000 days of MAs use. The median time from MA application to MARSI occurrence was 2.78 (min‐max, 1–8.6) days. The incidence rate of MARSI is presented in Table 2.

**TABLE 2:** The incidence rate and characteristics of MARSI.

Variables	Statistics
Total patients, *n*	72
Total MAs, *n*	302
Total days of MAs	1869
Incidence of MARSI	
Number of MARSIs occurred	44 cases
Incidence rate per 100 patients	61.1 cases
Incidence rate per 100 MAs	14.6 cases
Incidence rate per 1000 days of MAs	23.5 cases
Characterictics of MARSI, *n* (%)	140 (100%)
Skin stripping	101 (72.1%)
Tension injury	19 (13.6%)
Skin tears	14 (10%)
Dermatitis	6 (4.3%)
Time to MARSI occurrence, day	
Mean ± SD	3.02 ± 1.54
Median (min–max)	2.78 (1–8.6)

Abbreviations: MARSI, medical adhesive‐related skin injury; MAs, medical adhesives.

### 3.3 Characteristics of MARSI

**FIGURE 2:**
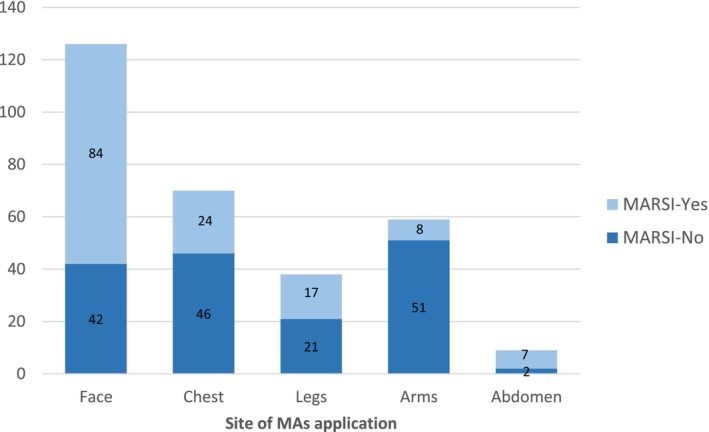
Number of MARSI by site.

**FIGURE 3:**
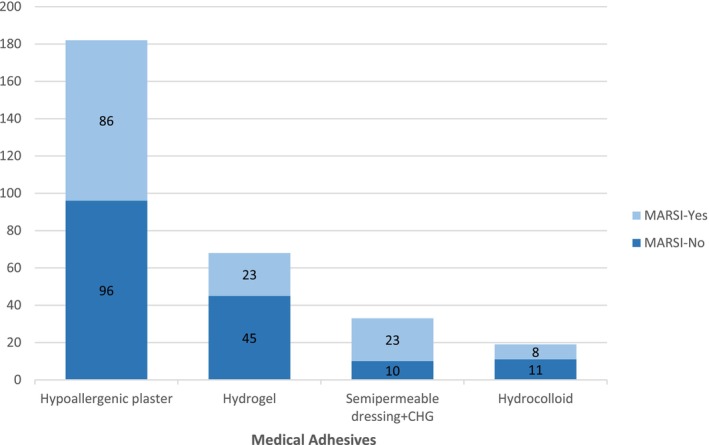
Number of MARSI by type of medical adhesives. CHG, chlorhexidine gluconate.

**FIGURE 4:**
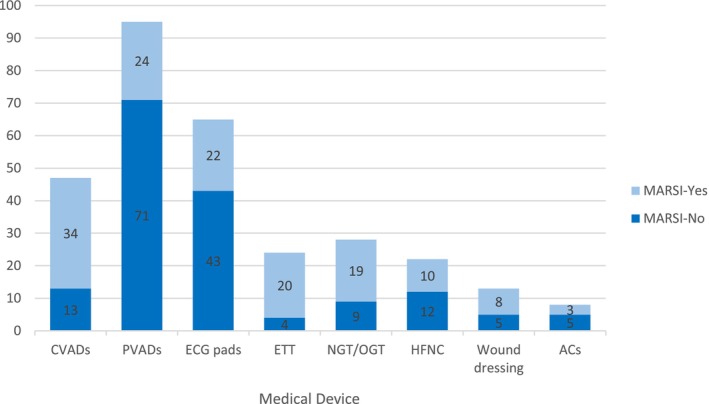
Number of MARSI by medical device. AC, artery catheter; CVADs, central venous access devices; ECG pads, electrocardiogram pads; ETT, endotracheal tube; HFNC, high flow nasal cannula; NGT, nasogastric tube; OGT, orogastric tube; PVADs, peripheral venous access devices.

Most of the MARSIs (*n* = 101; 72%) were skin stripping. The characteristics of MARSI are presented in Table 2. The highest rate of MARSI formation (*n* = 84; 67%) was found on the face (Figure 2). A total of 302 medical adhesives (MA) were applied to the patients. When the number of MARSI according to the type of MA was analysed, it was seen that 86 of 140 skin injuries were associated with hypoallergenic plaster, 23 with hydrogel (affix with ECG pad), 23 with semipermeable dressing with chlorhexidine gluconate (CHG) and 8 with hydrocolloid (Figure 3). The number of MARSI according to the number of medical devices were central venous access device (CVADs) (34/47), peripheral venous access devices (PVADs) (24/95), electrocardiogram pad (ECG pads) (22/65), ETT (20/24), nasogastric tube (NGT)/orogastric tube (OGT) (19/28), high‐flow nasal cannula (HFNC) (10/22), wound dressing (8/13) and artery catheter (3/8) (ACs) (Figure 4).

### 3.4 Associated Risk Factors of MARSI

When comparing the independent risk factors in patients with and without MARSI, univariate analysis results showed that being younger than 12 months, increased duration of PICU stay, receiving ventilatory support, not receiving oral nutrition, being sedated for more than 24 h, being immobilized and having a higher number of medical devices were significantly associated with the development of MARSI (*p* < 0.05). The other variables (sex, body mass index [BMI], diagnosis, presence of comorbidities, use of vasopressors, use of corticosteroids, laboratory findings and Glamorgan Risk Assessment Score) were not significantly associated with the development of MARSI (*p* > 0.05) (Table 3).

**TABLE 3:** Comparison of independent risk factors in patients with and without MARSI.

Parameters	Category	MARSI		*p*
		Yes (*n* = 44)	No (*n* = 28)	
Age, *n* (%)	< 12 months	25 (78.1)	7 (21.9)	0.008^a^ ^,^ *
	≥ 12 months	19 (47.5)	21 (52.5)	
Sex, *n* (%)	Male	21 (52.5)	19 (47.5)	0.094^a^
	Female	23 (71.9)	9 (28.1)	
BMI, mean (SD)	All	15.5 (4.9)	15.9 (3.2)	0.56^a^
Hospital stay (day), mean (SD)	All	9.7 (5.7)	5.5 (2.8)	< 0.001^a^ ^,^ *
Diagnosis, *n* (%)	RD	21 (60)	14 (40)	
	ND	8 (72.7)	3 (27.3)	
	GID	10 (66.7)	5 (33.3)	
	CD	1 (20.0)	4 (80)	
	CA	4 (66.7)	2 (33.3)	
Comorbidities, *n* (%)	Yes	17 (54.8)	14 (45.2)	0.342^a^
	No	27 (65.9)	14 (34.1)	
Respiratory support, *n* (%)	Yes	32 (72.7)	12 (27.3)	0.011^a^ ^,^ *
	No	12 (42.9)	16 (57.1)	
Feeding, *n* (%)	Oral	9 (37.5)	15 (62.5)	0.003^a^ ^,^ *
	Enteral	9 (100)	0 (0)	
	IV Fluid	26 (66.7)	13 (33.3)	
Use of sedation, *n* (%)	≤ 24 h	6 (24)	19 (76)	< 0.001^a^ ^,^ *
	> 24 h	38 (80.9)	9 (19.1)	
Use of vasopressor medications, *n* (%)	Yes	7 (77.8)	2 (22.2)	0.467^c^
	No	37 (58.7)	26 (41.3)	
Use of corticosteroids, *n* (%)	Yes	16 (59.3)	11 (40.7)	0.803^a^
	No	28 (62.2)	17 (37.8)	
C‐reactive protein, mean (SD)	All	42.7 (85.3)	50.0 (77.1)	0.481^b^
Haemoglobin, mean (SD)	All	10.3 (1.3)	10.1 (1.3)	0.57^b^
Albumin, mean (SD)	All	3.4 (0.6)	3.6 (0.5)	0.147^b^
PH, mean (SD)	All	7.3 (0.5)	7.4 (0.1)	0.548^b^
Mobilization status, *n* (%)	Immobilized	24 (52.2)	22 (47.8)	0.039^a^ ^,^ *
	Mobile/Semi‐Restricted	20 (76.9)	6 (23.1)	
Glamorgan score, mean (SD)	All	22.9 (7.4)	19.9 (5.7)	0.073^a^
Number of medical devices, mean (SD)	All	4.7 (1.1)	3.4 (1.0)	< 0.001^a^ ^,^ *

*Note:* The values are the means ± SDs or *n* (%).

Abbreviations: CA, cancer; CD, cardiovascular diseases; GID, gastrointestinal diseases; ND, neurological diseases; RD, respiratory disease; SD, standard deviation.

* *p* < 0.05.

^a^ Pearson chi‐square.

^b^ Mann–Whitney *U* test.

^c^ Fisher's exact test.

Multivariate logistic regression analysis identified that age 12 months or younger [OR = 5.3 (95% CI = 1.6–17.6); *p* = 0.006], increasing number of medical devices [OR = 2.4 (95% CI = 1.1–5.2); *p* = 0.026] and sedation use for more than 24 h [OR = 5.4 (95% CI = 1.7–16.8); *p* = 0.003] were independent risk factors for MARSI (Table 4).

**TABLE 4:** Independent variables associated with MARSI.

Variables	OR [95% CI]	*p*
Age (< 12 months vs. ≥ 12 months^a^)	5.323 [1.612–17.576]	0.006*
Number of medical devices	2.409 [1.111–5.223]	0.026*
Use of sedation (> 24 h vs. ≤ 24 h^a^)	5.407 [1.745–16.755]	0.003*
Constant	0.004	0.006

Abbreviations: CI, confidence interval; OR, odds ratio.

* *p* < 0.05.

^a^ Reference value.

**FIGURE 5:**
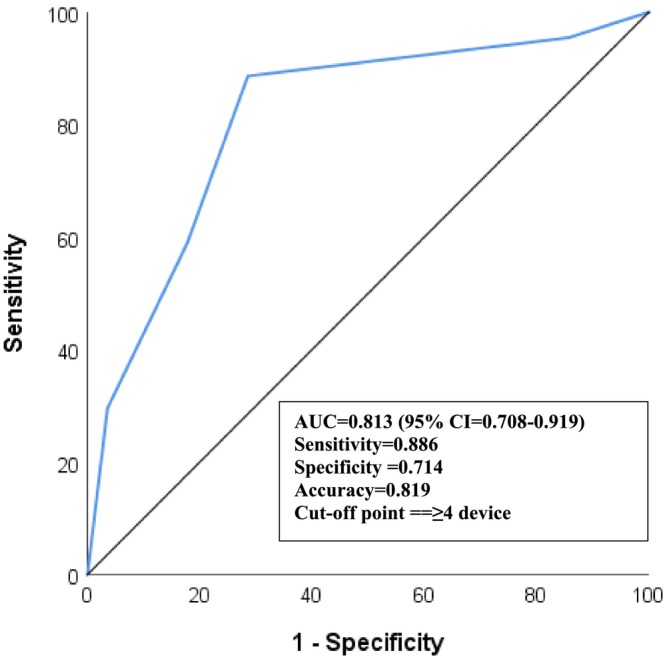
ROC curve.

The ROC analysis revealed that the optimal number of medical devices to determine the presence of MARSI was 4 [AUC = 0.81 (95% CI = 0.71–0.92); *p* < 0.001]. The sensitivity, specificity and accuracy rates for a limit of 4 medical devices were calculated as 89%, 71% and 82%, respectively (Figure 5).

## 4 Discussion

Unlike other clinical settings, treatment and care in the PICU requires many invasive interventions, which increases the use of medical adhesives and the risk of developing MARSI. In this study, the prevalence of MARSI was 61.1%. Similar to the results of this study, Crespo et al. reported an incidence of MARSI of 60.3% [8], Kim et al. reported 58.3% [7] and Wang et al. reported that this rate can be as high as 54.17%, with an average rate of 37.15% [4]. This study identified 23.5 cases of MARSI per 1000 days of medical adhesive use, a rate comparable to a study conducted in a neonatal intensive care unit, which reported 30 cases per 1000 days of medical adhesive use [16]. MARSI is recognized as a more prevalent complication in intensive care settings, particularly among paediatric patients [3, 7, 8, 11, 17]. The high incidence of MARSI in these populations is attributed to the absence of routine skin assessments specifically for MARSI and the lack of standardized methods for the application and removal of medical adhesives.

The findings of this study revealed that the median time from the application of medical adhesives to the development of MARSI was 2.78 days, with a range of 1 to 8.6 days. Similarly, Crespo et al. [8] reported that MARSI typically develops within the first 3 days of medical adhesive application in paediatric cardiovascular surgery intensive care units. MARSI is known to develop even more rapidly in neonates due to their skin structure and vulnerability [3, 8, 11, 16]. In infants, the elastic fibres in the dermis are not yet fully matured, leading to weaker cohesion between the dermis and epidermis compared with adults. This structural immaturity significantly heightens their risk of skin injuries caused by medical adhesive use [16, 18]. Nearly half of the patients in the present study were under the age of one, an important risk factor in understanding their increased susceptibility to MARSI.

The findings of the present study revealed that skin stripping was the most commonly observed type of MARSI (72%), followed by tension injury (14%), skin tears (10%) and contact dermatitis (4%). Similar to these results, studies conducted in PICUs in China and Korea have also reported skin stripping as the most frequently encountered type of MARSI [4, 7]. However, in a study conducted in a PCICU in Brazil, skin tears were identified as the most prevalent MARSI type [8]. It is important to note that the MARSI classification defined by the MARSI Consensus [11] was not utilized in this study [8]. As a result, it remains unclear whether injuries categorized as skin stripping were included in their findings. This discrepancy highlights the significance of employing standardized classifications for MARSI to ensure consistency and comparability across studies.

The findings of this study indicate that MARSI most commonly develops in the facial region, including the neck (Figure 2). Consistent with these results, previous studies have also reported that MARSI is most frequently observed in the facial area [3, 4, 7]. The vulnerability of the facial region to adhesive‐related injuries can be attributed to the thinner and more delicate skin compared with other parts of the body, making it more prone to damage. Furthermore, the frequent use of medical adhesives in this area during procedures such as intubation, feeding, central venous catheterization or monitoring significantly increases the risk of skin injuries.

In the clinic where this study was conducted, hypoallergenic plaster is used to secure peripheral venous catheters (PVCs), ETTs and OGT/NGT to the skin. Additionally, some CVADs are also fixed using hypoallergenic plaster. This widespread use of hypoallergenic plaster explains the high number of MARSI cases observed in this study (Figure 3).

However, when the MARSI cases are analysed based on the type of MAs, the number of MARSI cases associated with the use of semipermeable dressings with chlorhexidine gluconate CHG is also notably high (Figure 3). These dressings are primarily used to secure CVADs. Furthermore, when MARSI cases are evaluated according to the medical device used, the findings clearly show that MARSI occurrence is significantly higher in patients with CVADs (Figure 4). These findings are consistent with studies in the literature reporting high rates of MARSI associated with CVADs, particularly when secured to the skin with semipermeable dressings that have strong adhesive properties and require frequent adhesive changes [2, 4, 5, 19, 20].

Medical adhesives are widely used for a variety of purposes in intensive care settings [1, 2]. Moreover, the adhesive strength of each product varies depending on its material composition. Therefore, to prevent MARSI, it is crucial to carefully select the adhesive type based on the application site and whether the medical device is critical. Additionally, applying and removing adhesives using proper techniques plays a significant role in minimizing the risk of MARSI [10, 16, 21].

The results of the present study showed that being younger than 12 months is associated with an increased risk of developing MARSI in paediatric patients. Consistent with our findings, younger age has been linked to a higher risk of MARSI due to the immaturity of the skin [1, 10, 11, 16].

Additionally, prolonged PICU stays, the need for respiratory support, a higher number of medical devices and immobilization were identified as factors associated with MARSI development. Recent studies have highlighted that as the length of hospital stay increases, mobilization decreases and the use of adhesives to secure life‐support and monitoring devices, such as those used for respiratory support, becomes more frequent, thereby increasing the risk of MARSI [2, 16].

These findings were further confirmed through ROC analysis, which strikingly demonstrated that the use of four medical devices is the most suitable threshold for determining the risk of MARSI. Supporting our results, Crespo et al. [8] also reported a strong association between an increased number of medical devices and the development of MARSI.

This study found that sedation for more than 24 h and the inability to receive oral nutrition are linked to a higher risk of MARSI. It is well known that nutritional support plays a crucial role in maintaining skin integrity and facilitating tissue repair [1]. PICUs, where complex treatments are utilized, are high‐risk environments. While sedation can lead to hypotension and vasodilation, the relationship between sedation and MARSI risk remains unclear [8].

## 5 Strengths and Limitations

One of the strengths of this study is the use of the MARSI classification, which is not commonly utilized in most studies on MARSI. The absence of such classification in other studies complicates the determination of the type and severity of MARSI. Another strength is the application of multivariable analysis to identify independent risk factors for MARSI. Additionally, the study employed ROC analysis to estimate a threshold for the number of medical devices associated with MARSI development. However, a limitation of this study is the small sample size and single‐centre design. To enhance the accuracy of MARSI incidence rates and associated risk factors, we strongly recommend conducting multicentre studies with larger sample sizes.

## 6 Implications and Recommendations for Practice

Maintaining skin integrity is a key indicator of the quality of nursing care. MARSI, a highly common yet preventable complication, is influenced by numerous factors. The findings of this study can help raise nurses' awareness of the incidence, characteristics and risk factors of MARSI in PICU settings, contributing to the development of improved care approaches.

Children are more vulnerable to skin injuries than adults and these injuries can develop rapidly. Therefore, nurses need to be careful in the selection, application and removal of medical adhesives. The development of evidence‐based care bundles aimed at preventing MARSIs may be an effective solution to reduce their incidence and improve quality of care.

## 7 Conclusion

The incidence of MARSI is high in children in the PICU. The most commonly seen classification of MARSI is skin stripping, and the most affected area is the face. Being younger than 12 months, receiving prolonged sedation for more than 24 h, and increased use of medical devices are risk factors for MARSI. These findings underscore the critical role of nurses in preventing MARSI through evidence‐based practices, such as care bundles that include routine skin assessments, appropriate selection of adhesives and correct application and removal techniques, as well as adherence to clinical guidelines.

Raising awareness and enhancing knowledge through regular training programmes for PICU nurses are essential steps in reducing MARSI frequency. By integrating these preventive strategies into daily care, nurses can significantly provide improved patient outcomes and maintain skin integrity in paediatric patients.

## Ethics Statement

The study was approved by the Ümraniye Training and Research Hospital Ethics Committee (Date: November 2, 2023, Reference No: B.10.1.TKH.4.34.H.GP.0.01/435). Additionally, written institutional permission was obtained from the institution where the research was conducted. The research was conducted in accordance with the principles of the Helsinki Declaration.

## Consent

Parents or guardians of children included in the study were informed about the research, voluntary informed consent forms were signed and consent was obtained from applicable children.

## Conflicts of Interest

The authors declare no conflicts of interest.

## Data Availability

The data that support the findings of this study are available from the corresponding author upon reasonable request.
